# Retraction: IL-21 Regulates the Differentiation of a Human γδ T Cell Subset Equipped with B Cell Helper Activity

**DOI:** 10.1371/journal.pone.0223172

**Published:** 2019-09-24

**Authors:** The PLOS ONE Editors

Following publication of this article [1], concerns were raised about the flow cytometry image data in Figures 1 and 3. Specifically,

- In Figure 1A, there are regions of similarity between the CD27 positive/ICOS negative quadrant in the Nil panel and the CD27 negative/ICOS negative quadrant in the IL-2 panel.
- In Figure 1A, there are regions of similarity between the CD27 positive/ICOS negative quadrant of the IL-15 panel and the CD27 positive/ICOS negative quadrant of the IL-21 panel when the quadrant is flipped horizontally.
- In Figure 1C, the CD40L histogram panel is similar to the ICOS histogram panel.
- In Figure 3A, the right-hand slope on the Ag histogram is similar to the right-hand slope on the DC histogram.

The authors have provided the individual-level data underlying the charts in Figures 1 and 3. The corresponding author has stated that the service facility at the University Hospital in Palermo performed the FACS analyses and provided printed FACS plots, and the underlying flow cytometry data files are not available.

Owing to the unavailability of the underlying flow cytometry data, the above issues could not be resolved. In light of the concerns regarding the preparation of Figures 1A, 1C and 3A and the handling of flow cytometry data, the *PLOS ONE* Editors retract this article.

NC, MPLM, Guido Sireci, FD did not agree with retraction. MT did not respond. Giorgio Stassi did not comment on the retraction decision.
